# Influence of human intervention on rodent population dynamics in Southwest China

**DOI:** 10.1016/j.onehlt.2025.101276

**Published:** 2025-11-14

**Authors:** Wei Zhao, Yuqiong Li, Dongdong Lin, Jing Lu, Zhe Lou, Xiyang Li, Zhengxiang Liu, Siyu Li, Jian Wang, Rongji Cao, Zihou Gao, Zongti Shao, Ruiyun Li

**Affiliations:** a School of Public Health, Nanjing Medical University, Nanjing, Jiangsu 211166, China; b Yunnan Institute of Endemic Diseases Control and Prevention, No. 5 Wenhua Road, Dali, Yunnan, China; c Key Laboratory of Modern Toxicology, Ministry of Education, Nanjing, Jiangsu 211166, China; d Provincial University Key Laboratory of Public Health Safety and Emergency Prevention and Control Technology, Nanjing, Jiangsu, 211166, China

**Keywords:** Rodent population density, Wild plague foci, Monitoring, Human intervention

## Abstract

Rodents are major reservoirs of zoonotic pathogens, presenting substantial public health threats in plague-endemic regions. Although chemical rodenticides have been widely used to control populations to reduce the risk of infectious diseases and epidemics, their long-term effectiveness and ecological impacts under diverse environmental conditions remain poorly understood. Here, we conducted a longitudinal field study across eight sites in Jianchuan and Yulong counties (Yunnan Province, China) from April 2023 to April 2024, to evaluate control effects and strategies by comparing rodent population dynamics in intervention and non-intervention areas. Our results showed that rodenticide effectively reduced rodent density in the short term, but population recovery exhibited strong spatial variation, driven by ecological conditions and intervention strategies. Statistical modelling further identified leaf area index (LAI) of low vegetation, soil temperature, and soil moisture content as key predictors of rodent population resurgence. These results emphasize the spatially heterogeneities effectiveness of rodenticides and highlight the influence of environmental conditions on post-intervention population recovery. Our study advocates for ecologically adaptive rodent management strategies to enhance the sustainability and precision of zoonotic disease control in endemic regions.

## Highlights

• Rodent study took place in two Yunnan counties over one year.
• Rodenticide cut rodent numbers fast but recovery differed between regions.
• The study shows local setting shapes how well rodent control works.

## 1 Introduction

Rodents represent the most diverse mammalian order, comprising over 2500 species (∼42 % of global mammal biodiversity) [1,2] and exhibiting remarkable ecological adaptability [2] due to high reproduction rates and short gestation periods of rodents [3]. While rodents are key species in ecosystems as prey and seed dispersers [4], they also serve as reservoirs for more than 85 pathogens [5]. Historical records of human infections with rodent-borne diseases, including plague caused by *yersinia pestis*, hantavirus, and typhus, highlight that rodents are critical reservoirs for diseases among human populated areas [[6], [7], [8]]. Meanwhile, rodent population fluctuations are closely associated with the emergence and intensity of rodent-borne zoonotic disease outbreaks [9]. Understanding how human interventions alter rodent population dynamics is thus fundamental for mitigating public health risks.

As human plague outbreaks typically result from the spillover of animal epizootics, chemical rodenticides that have long been regarded as a primary strategy for reservoir control globally exhibit variable efficacy and ecological impact across empirical settings [10]. This discrepancy may be partially attributable to the doses of rodenticide and insecticides. For example, high concentrations of anticoagulant rodenticides such as bromadiolone can induce bait shyness or sub-lethal exposure, potentially resulting in behavioral avoidance and reduced efficacy over time [11]. Furthermore, population recovery post-intervention may be influenced by local climatic conditions, such as temperature and precipitation [4], well as immigration from other populations. However, these interactions remain poorly quantified under real-world contexts.

Yunnan Province, a region of exceptional biogeographic complexity characterized by steep elevational gradients, diverse climate zones, and heterogeneous habitats from tropical lowlands to alpine regions [12], exemplifies these challenges. Such ecological environment allows the survival of rodents and the transmission of associated diseases [13]. Notably, Yunnan was identified as the origin of the third global plague pandemic and still contains two distinct natural plague foci [14]. The continued existence of these enzootic areas contribute to the cyclical nature of plague outbreaks and underscore the enduring complexity of plague control. Despite the success of China's Patriotic Health Campaign in eliminating the human plague in 1956 [15], zoonotic plague persists in rodent reservoirs, with re-emergence reported since 1982 [16]. Moreover, in ecologically undisturbed or semi-managed regions, such as the Yunnan Plateau, plague circulates among rodent populations and their flea vectors [17,18,19]. Sylvatic plague cycles in undisturbed areas underscore the need for evidence-based and sustainable management of rodent-borne diseases.

In this study, we quantified the efficacy of bromadiolone rodenticides across Yunnan’ wild plague foci, monitoring rodent population dynamic from April 2023 to April 2024 under varying baits formulations and environmental conditions. By integrating ecological variables (e.g., vegetation cover and microclimate), we further explored the interplay between anthropogenic interventions and environmental factors of population recovery. Our findings provide evidence to guide the development of more targeted and ecologically informed strategies for rodent-borne disease control in endemic regions.

## 2 Methods

### 2.1 Study design

**Fig. 1:**
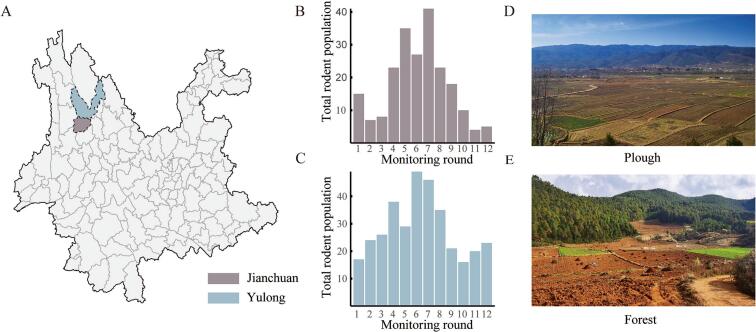
Distribution of rodent populations across Jianchuan and Yulong regions. (A) Map of the study area, showing the locations of Jianchuan and Yulong. (B) Distribution of total rodent population in Jianchuan by monitoring round. (C) Distribution of total rodent population in Yulong by monitoring round. (D) Representative plough habitat in Jianchuan. (E) Representative forest habitat in Yulong.

The study was conducted in Jianchuan and Yulong counties in Yunnan Province, China, selected based on their history of plague activity and distinct ecological characteristics [20,21]. Jianchuan County features a subtropical highland climate with predominantly agricultural landscapes including crop fields, rural settlements, and fragmented shrublands (Fig. 1D). In contrast, Yulong County has a temperate alpine climate characterized by mixed forest-agricultural mosaics (Fig. 1E), including subalpine coniferous forests and grasslands that support diverse small mammal communities [22]. These features allow comparative analyses for evaluating the impact of intervention strategies and environmental factors on rodent population. We placed four surveillance sites in each county: Daqing A, B, C and D in Jinhua Town (Jianchuan), and Jizi A, C, Tianhong B and D in Tai'an Town (Yulong). Among these sites, Daqing D and Tianhong D served as untreated control sites, while the remaining sites received one of three rodenticide treatments: 0.005 % bromadiolone pellets, 0.01 % toxic rice bait (bromadiolone-based), and 0.01 % bromadiolone pellets. Interventions were applied only after the first monitoring round, ensuring that baseline rodent populations were established prior to treatment application.

### 2.2 Rodent population monitoring

Monthly surveillance of rodents was performed from April 2023 to April 2024 following the guidelines outlined in National Plague Monitoring Programme and Yunnan Province Plague Monitoring Programme. Baseline rodent density was assessed in April 2023 using the cage-night method [23] before implementing interventions. To avoid introducing human-induced biases during the population recovery phase, all captured rodents were identified, recorded, and released at the point of capture. This monitoring approach was designed to minimize ecological disturbance and prevent interference with repopulation dynamics, thereby ensuring the scientific rigor of the recovery evaluation. Following this baseline assessment, the intervention groups were exposed to different concentrations and types of rodenticide treatments, while the control group did not impose human intervention and maintained routine monitoring protocols. One month after the implementation of the intervention, 100 rodent traps were systematically deployed at each monitoring site-each covering approximately 4 to 6 ha-along ridges, ditches, and other representative habitats, with 5 m spacing between traps and ≥ 20 m between rows. Cages were set in the evening and inspected the following morning to record capture rates, species composition, and other relevant ecological data. An effective rat cage was defined as a trap that was successfully retrieved and functional, excluding any that were lost or damaged during the trapping period. “Recovery” refers specifically to the process of the rodent population density returning to its pre-treatment (baseline) level after the rodenticide intervention [24]. For rodents captured and transported to the laboratory, both the animal and the trap were immediately placed in an individual bag to contain and collect any fleas that detached from the host during transit.

### 2.3 Environmental data collection

Environmental variables including air temperature at 2 m height, total precipitation, leaf area index (LAI), soil temperature, and soil volumetric water content were obtained from the fifth-generation reanalysis (ERA5) provided by the European Center for Medium-Range Weather Forecasts (ECMWF) [25]. Daily data were extracted for the grid point nearest to each surveillance site and aggregated to generate township-level environmental metrics.

### 2.4 Statistical analyses

Rodent density (R) was defined as the number of rodents captured per effective trap using R=Nr/Ne×100%, where Nr and Ne is the number of rodents captured and the number of effective rat cages (traps), respectively. Relative rodent density (RRD) was computed as the difference between capture rates at time t and baseline (${\mathrm{RRD}}_{t}={\mathrm{CR}}_{t}-{\mathrm{CR}}_{\mathrm{baseline}}$). Initial data exploration employed descriptive statistics and independent samples *t*-tests to compare rodent populations between counties. Temporal population dynamics were analyzed using linear regression and B-spline smoothing. The influence of meteorological and environmental factors on population recovery was assessed through Generalized Additive Models (GAMs) incorporating smooth terms for nonlinear relationships:

$$\log\left(\frac{Y_{i}}{1-Y_{i}}\right)=\alpha_{i}+\gamma C_{j}+s\left(\mathrm{LA}I_{i}\right)+s\left(ST_{i}\right)+s\left(SM_{i}\right)+s\left(AT_{i}\right)+s\left(TP_{i}\right)$$

where $Y_{i,j}$ represents rodent density on day i in region j; $\alpha_{i,j}$is the intercept, and $\gamma C_{j}$ denotes rodenticide treatment effects. $s\left(\right)$are smooth functions for leaf area index (LAI), soil temperature (ST), soil volume of water (SW), air temperature (AT), and total precipitation (TP) on rodent population recovery. All analyses were conducted using Microsoft Excel 2019 and R Statistical Software (v4.3.3; R Core Team) in RStudio, with statistical significance set at *p* < 0.05.

## 3 Results

### 3.1 Rodent population dynamics without interventions

During the 2023–2024 surveillance period, we captured a total of 2358 rodents from 9951 trap-nights across eight sites in Yulong and Jianchuan counties, yielding an overall capture rate of 23.7 % (Fig. 1A, Table 1). The rodent community was dominated by *Apodemus chevrieri*, which accounted for 92.4 % of total captures. This dominance of *Apodemus chevrieri* was observed in both counties. More specifically, 90.5 % (1009 out of 1115) of the captured rodents were *Apodemus chevrieri* in Jianchuan; while around 95 % (1170 out of 1243) rodents were *Apodemus chevrieri* in Yulong (Table 1). Control sites exhibited clear seasonal population fluctuations (Fig. 1B-C), with significantly higher baseline densities in Yulong compared to Jianchuan (Fig. S1). The total rodent population peaked between monitoring rounds 5 and 7, followed by a gradual decline observed during rounds 10 to 12.

**Table 1:** Rodent capture statistics and intervention measures in Jianchuan and Yulong.

Epidemic Spot			Treatment	Number of rat cages (traps)	Number of captures	Capture rate/%	*Apodemus Chevrieri* Quantity	*Apodemus Chevrieri* Capture rate/%
County	Township	Village						
Jianchuan	Qinghua	Daqing A	0.005 % bromadiolone formulated pellets	1216	307	3.1 %	296	3.0 %
		Daqing C	0.01 % bromadiolone formulated pellets	1229	299	3.0%	256	2.6 %
		Daqing B	0.01 % prepared toxic rice bait	1230	293	2.9 %	278	2.8 %
		Daqing D	Control	1335	216	2.2 %	179	1.8 %
Yulong	Taian	Jizi A	0.005 % bromadiolone formulated pellets	1222	335	3.4 %	319	3.2 %
		Jizi C	0.01 % bromadiolone formulated pellets	1251	299	3.0%	276	2.8 %
		Tianhong B	0.01 % prepared toxic rice bait	1234	265	2.7 %	249	2.5 %
		Tianhong D	Control	1234	344	3.5 %	326	3.3 %
Total				9951	2358	23.7 %	2179	21.9 %

### 3.2 Effects of rodenticide interventions

**Fig. 2:**
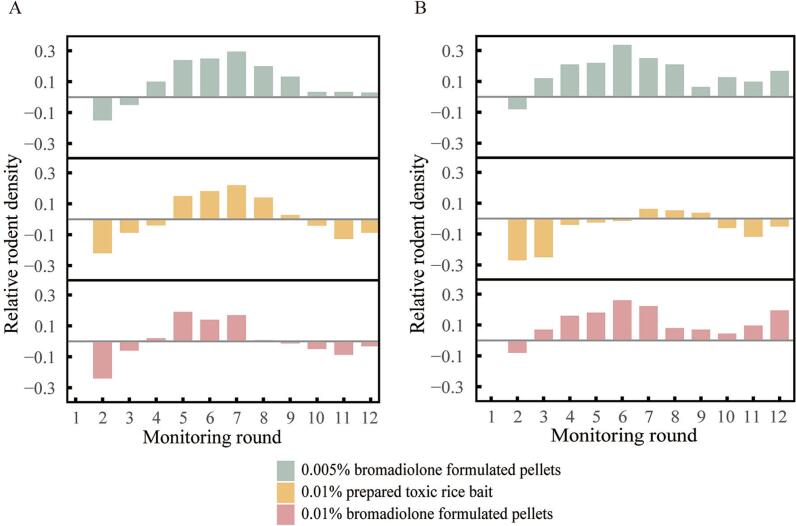
Temporal dynamics of relative rodent density across different treatments. (A) Rodent density changes for three intervention groups in Jianchuan: 0.005 % bromadiolone formulated pellets, 0.01 % prepared toxic rice bait, and 0.01 % bromadiolone formulated pellets. (B) Rodent density changes for the same treatments in Yulong. Data are presented by monitoring round, with relative rodent density shown on the vertical axis.

All three rodenticide treatments resulted in notable reductions in rodent density, with relative rodent density (RRD) values in the first post-intervention monitoring round decreased by 0.08 to 0.27 compared to baseline levels (Fig. 2, Fig. S2). However, the duration of population suppression varied substantially between treatments. Our surveillance showed that interventions using 0.01 % prepared toxic rice bait was the most sustained approach, maintaining reduced rodent densities for 3–4 months. In contrast, concentrations of bromadiolone bait (0.005 % and 0.01 %) showed short-term efficacy, with population recovery occurring within 1–2 months.

**Fig. 3:**
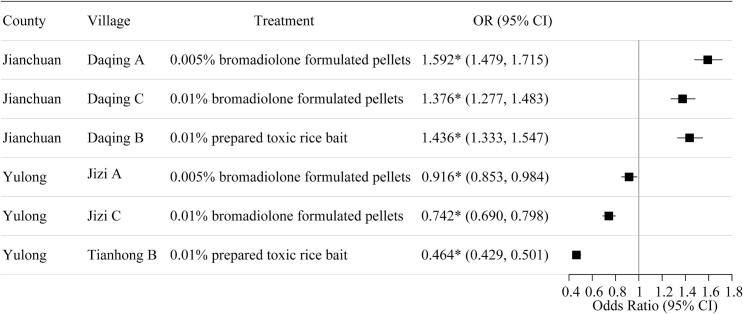
Estimated effects of rodenticide treatments on rodent density recovery. Odds ratios (ORs) and 95 % confidence intervals (CIs) for rodent density recovery under different rodenticide treatment conditions across two plague-endemic regions. Each row represents a specific treatment applied in a given village. Asterisks (*) indicate statistical significance (*p* < 0.05).

Notably, we observed striking regional differences in treatment outcomes between Jianchuan and Yulong counties (Fig. 3). In Jianchuan, all rodenticide treatments were associated with accelerated population recovery compared to control sites, with the 0.005 % bromadiolone bait treatment showing the strongest effect (OR = 1.592, 95 % CI: 1.479–1.715). Conversely, treatments in Yulong significantly suppressed population recovery, particularly with the 0.01 % prepared toxic rice bait, which reduced the predicted density to approximately 46.4 % of control levels (95 % CI: 0.429–0.501). These findings underscore regional differences in rodenticide efficacy and population rebound dynamics (Fig. 3). These results suggest that intervention effectiveness varied substantially between regions, with Yulong showing more pronounced and longer-lasting suppression of rodent populations compared to Jianchuan.

### 3.3 Environmental influences on population dynamics

**Fig. 4:**
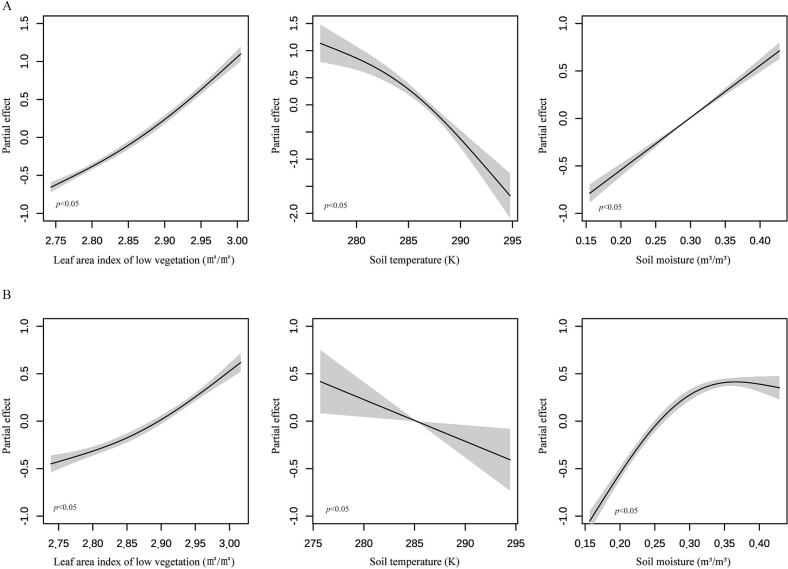
Climatic factors influencing rodent density recovery. (A) Partial effects of leaf area index (LAI) of low vegetation, soil temperature, and soil moisture on rodent density recovery were quantified using GAMs in Jianchuan. (B) Partial effects of leaf area index (LAI) of low vegetation, soil temperature, and soil moisture on rodent density recovery were quantified using GAMs in Yulong.

Our analysis identified three key environmental factors associated with rodent population dynamics (Fig. 4, Fig. S3). First, we found a significant non-linear relationship between LAI of low vegetation and rodent density, with higher leaf area LAI values generally corresponding to increased rodent populations. Additionally, soil water content showed significant association with rodent density in Yulong, where populations peaked at approximately 0.35 m [3]/m [3] volumetric water content before declining at higher moisture levels. This relationship was not observed in Jianchuan, suggesting site-specific environmental effects. Moreover, soil temperature showed a consistent linear association with rodent density across surveillance sites in two counties, with warmer soils generally supporting higher rodent populations. These findings highlight the complex interplay between environmental conditions and rodent population dynamics in these ecosystems.

## 4 Discussion

Our comparative study of rodent control interventions in Yunnan's plague-endemic regions demonstrates that while rodenticides effectively reduce rodent densities in the short term; however, their long-term efficacy varies substantially by formulation and local ecological context. The 0.01 % prepared toxic rice bait showed superior sustained suppression compared to bromadiolone pellets (0.005 % and 0.01 %), highlighting how bait selection critically influences intervention outcomes for zoonotic disease control.

The observed performance differences among rodenticides likely reflect variations in feeding behavior and toxicological effects [26]. The prepared toxic rice bait may exhibit higher palatability [27], increasing the likelihood of voluntary ingestion by the target population, thereby resulting in a higher effective dose and prolonged suppression of population rebound. In contrast, conventional toxic baits may induce neophobia or bait avoidance behavior [27], leading to sublethal intake levels in some individuals. This insufficient exposure can facilitate more rapid population recovery and ultimately compromise the overall efficacy of the intervention. High concentrations of bait compounds may cause secondary poisoning of predators and scavengers through biomagnification, thus posing a serious ecological risk [28]. A recent field study in New Zealand further demonstrated that combination baiting strategies can be effective in suppressing rodent populations [29]. Collectively, these findings underscore rodenticide type and concentration are critical determinants of suppression efficacy. Optimizing bait formulations and delivery strategies would be the key to achieving sustained rodent population control.

It is noteworthy that identical interventions produced divergent outcomes between Jianchuan and Yulong counties. The prolonged suppression in Yulong's alpine environment versus Jianchuan's agricultural landscape underscores how local ecology mediates control efficacy. Such spatial difference suggests that climatic conditions, vegetation cover, and habitat complexity may jointly influence rodent population dynamics. An increase in leaf area index of low vegetation reflects the enhanced coverage by herbaceous or dwarf shrubby plants, which provides rodents with more abundant shelter and food resources, thereby promoting higher reproductive rates and survival among host species [30,31]. Notably, local rodent populations (*Apodemus chevrieri*) tend to prefer ecotonal habitats, particularly bunds or field margins at the interface between farmland and forest, where such vegetation is typically dense and continuous. In addition, soil temperature was found to exert a significant influence on rodent population density. As many rodent species exhibit a burrow-dwelling lifestyle, their ecological behavior and physiological performance are closely tied to subterranean microclimatic conditions. Environmental changes, such as elevated soil temperatures, may induce thermal stress and elevate metabolic demands [32], ultimately reducing their survival and reproductive success. This process can partially suppress population recovery, indicating that soil temperature may serve as a critical environmental factor limiting rodent population expansion. These findings demonstrate how microclimate and habitat structure shape rodent demography, suggesting that environmental monitoring could help predict intervention success.

Seasonal reproduction and dispersal likely influenced post-intervention population recovery. In temperate small mammals, winter mortality and spring–summer breeding drive annual density fluctuations [33]. In our study areas, rodenticide application took place in early spring, just before the main breeding period, which may have delayed immediate population rebound. Nevertheless, recruitment and possible immigration from adjacent habitats could have contributed to the subsequent recovery observed later in the year. While our one-year dataset provides robust evidence for bait-specific effects, the evaluation of treatment effectiveness should be interpreted with some caution, as only one control site and a single replicate per rodenticide concentration were available in each region. This study design was influenced by the complex terrain, allocation of field personnel, and local rodent control regulations. Future work will aim to include additional replicates standardized concentrations and extend the monitoring duration to further strengthen the assessment of rodenticide efficacy under diverse ecological settings. An integrated approach combining chemical, ecological, and biological control strategies will be essential for the sustainable management of rodent populations. Future work should also evaluate cost-effectiveness and implementation barriers across different socioeconomic contexts in endemic regions.

## Ethics statements

The procedures and protocols for sample collection and processing in this study were reviewed and approved by the Medical Ethics Committee of the Yunnan Institute of Endemic Disease Control and Prevention.

## CRediT authorship contribution statement

**Wei Zhao:** Writing – original draft, Investigation, Formal analysis, Data curation. **Yuqiong Li:** Writing – original draft, Investigation, Data curation. **Dongdong Lin:** Data re-analysis, Investigation, Writing – review & editing. **Jing Lu:** Writing – original draft, Data curation. **Zhe Lou:** Methodology, Formal analysis. **Xiyang Li:** Methodology, Data curation. **Zhengxiang Liu:** Investigation. **Siyu Li:** Investigation. **Jian Wang:** Investigation. **Rongji Cao:** Investigation. **Zihou Gao:** Writing – review & editing, Supervision, Funding acquisition. **Zongti Shao:** Writing – review & editing, Supervision, Conceptualization. **Ruiyun Li:** Writing – review & editing, Supervision, Funding acquisition, Conceptualization.

## Declaration of competing interest

The authors declare that they have no known competing financial interests or personal relationships that could have appeared to influence the work reported in this paper.

## Data Availability

The datasets used and/or analyzed during the current study are available from the corresponding author on reasonable request.

## Appendix A Supplementary data

Supplementary material Image 1
