# Supplementary material for: Influence of human intervention on rodent population dynamics in Southwest China
Source: One Health. 2025 Nov 14;21:101276. doi: 10.1016/j.onehlt.2025.101276 (PMC12670526; doi:10.1016/j.onehlt.2025.101276)
Supplement: Supplementary file 1 — Supplementary material [file mmc1.docx]

**Table S1: Species composition of captured rodents in Jianchuan and Yulong**

| **County** | **Township** | **Species** | **Number of captures** | **Composition (%)** |
| --- | --- | --- | --- | --- |
| Jianchuan | Qinghua | *Apodemus chevrieri* | 1009 | 90.4% |
|  |  | *Rattus niviventer* | 75 | 6.7% |
|  |  | *Apodemus latronum* | 14 | 1.3% |
|  |  | *Apodemus draco* | 8 | 0.7% |
|  |  | *Other rodents* | 10 | 0.9% |
| Yulong | Taian | *Apodemus chevrieri* | 1170 | 94.1% |
|  |  | *Apodemus draco* | 26 | 2.1% |
|  |  | *Rattus tanezumi* | 23 | 1.9% |
|  |  | *Rattus niviventer* | 20 | 1.6% |
|  |  | *Other rodents* | 5 | 0.4% |

**
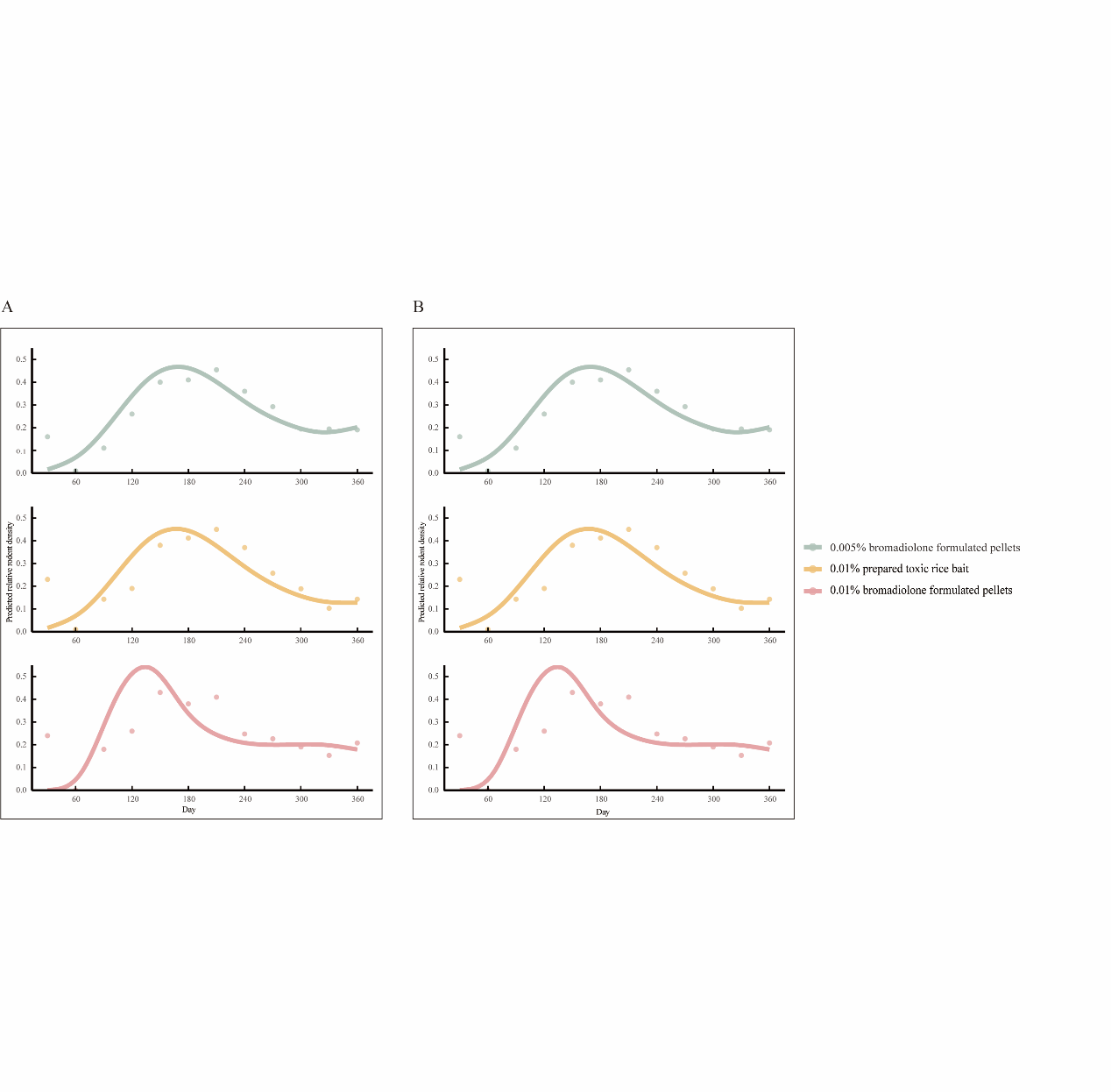
**

**Figure S1. Predicted relative rodent density over time under different rodenticide treatments.** The solid lines represent the model-predicted relative rodent density, while the scatter points indicate the observed values. (A) Predicted relative rodent density under three intervention measures in Jianchuan: 0.005% bromadiolone formulated pellets, 0.01% prepared toxic rice bait, and 0.01% bromadiolone formulated pellets. (B) Predicted relative rodent density under three intervention measures in Yulong: 0.005% bromadiolone formulated pellets, 0.01% prepared toxic rice bait, and 0.01% bromadiolone formulated pellets.

**
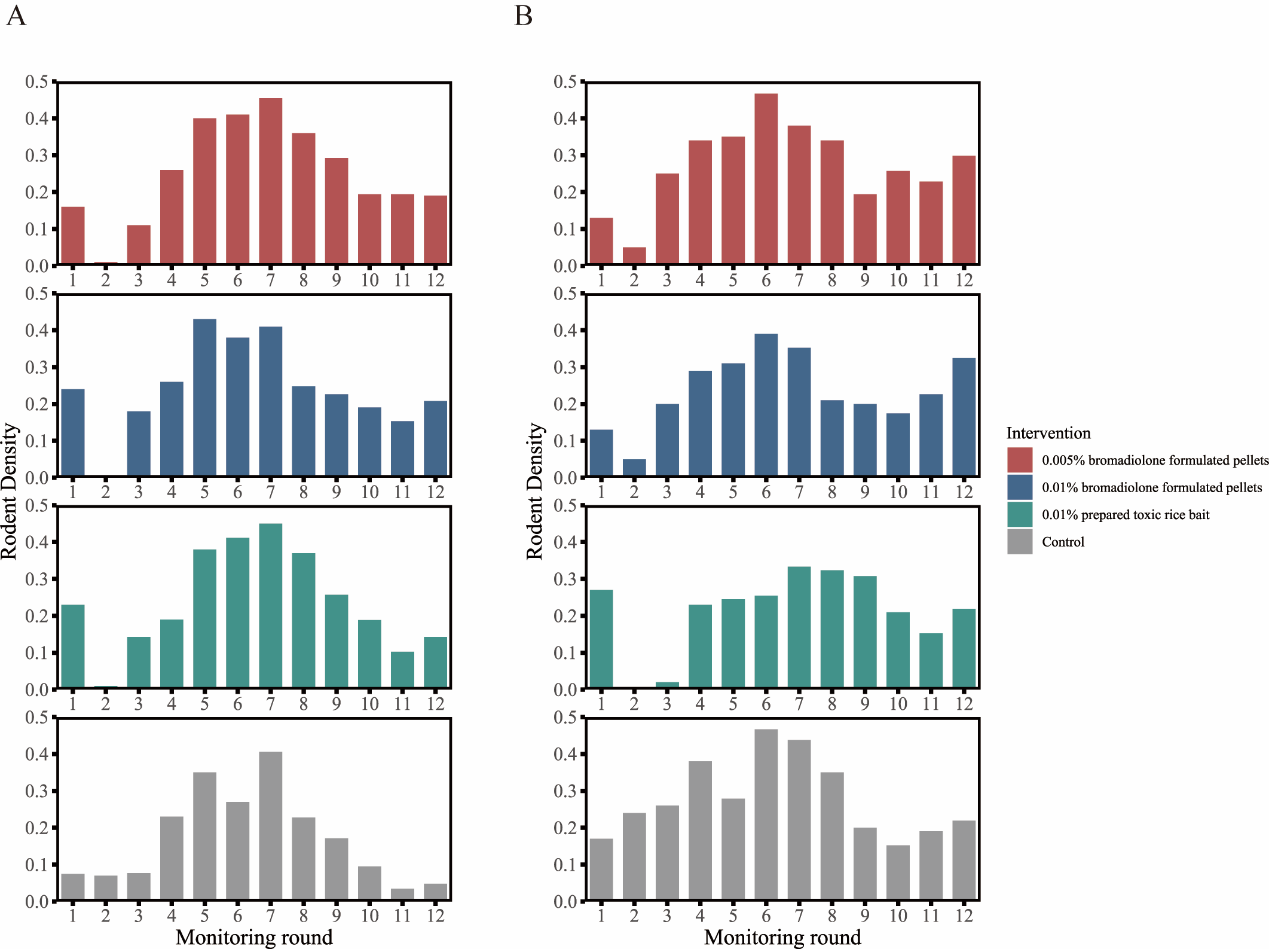
**

**Figure S2. Rodent population dynamics under different treatments in Jianchuan and Yulong.** (A) Temporal changes in total rodent population across different experimental treatments in Jianchuan. (B) Temporal changes in total rodent population across different experimental treatments in Yulong.

**
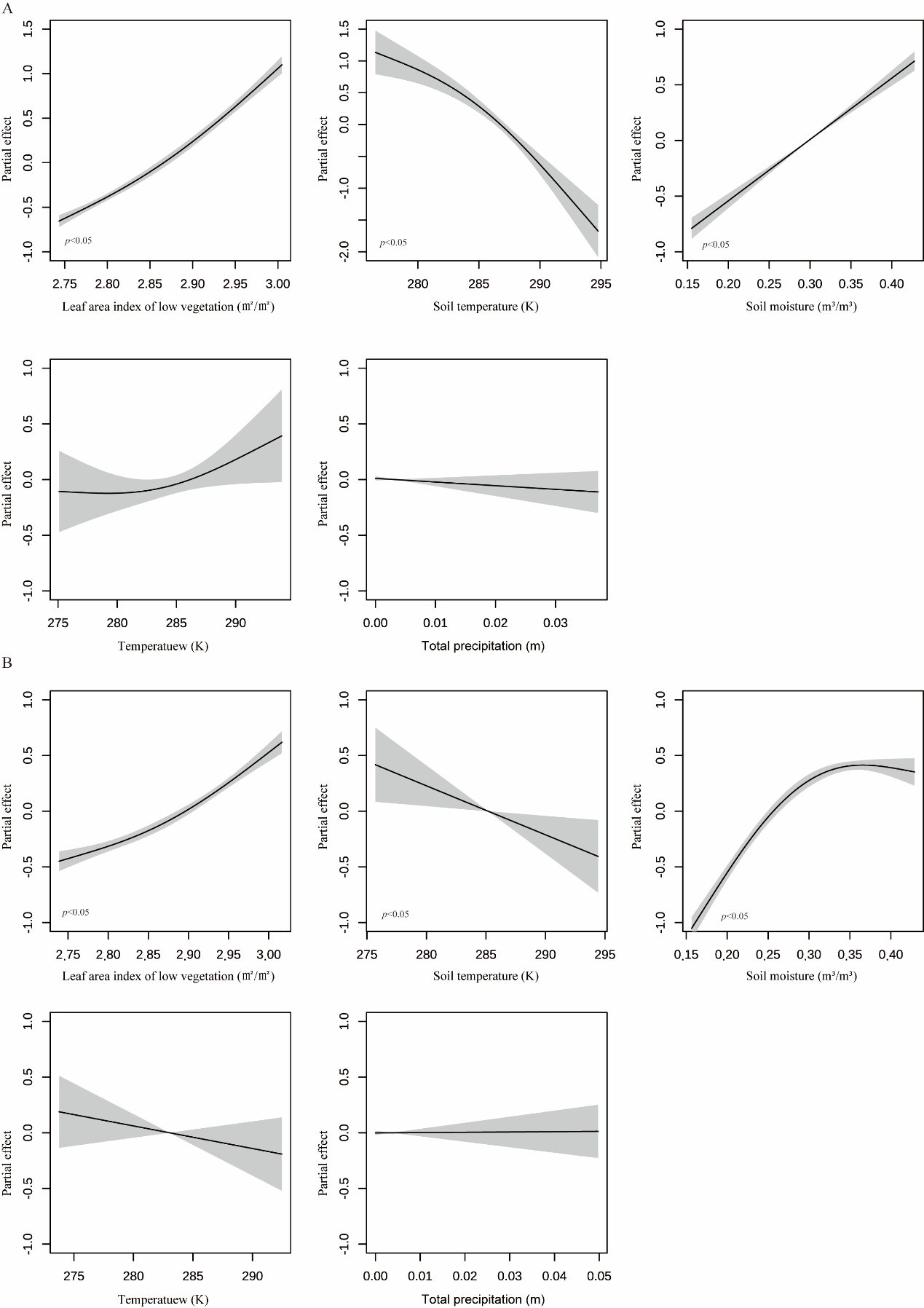
**

**Figure S3. Climatic factors influencing rodent density recovery** (A) Partial effects of leaf area index (LAI) of low vegetation, soil temperature, soil moisture, temperature and total precipitation on rodent density recovery were quantified using GAMs in Jianchuan. (B) Partial effects of leaf area index (LAI) of low vegetation, soil temperature, soil moisture, temperature and total precipitation on rodent density recovery were quantified using GAMs in Yulong.
